# H-Ras Exerts Opposing Effects on Type I Interferon Responses Depending on Its Activation Status

**DOI:** 10.3389/fimmu.2017.00972

**Published:** 2017-08-11

**Authors:** Guann-An Chen, Yun-Ru Lin, Hai-Ting Chung, Lih-Hwa Hwang

**Affiliations:** ^1^Institute of Microbiology and Immunology, National Yang-Ming University, Taipei, Taiwan

**Keywords:** H-Ras, type I interferon, retinoic acid-inducible gene-I-like receptor, MAVS signalosome, antiviral immunity

## Abstract

Using shRNA high-throughput screening, we identified H-Ras as a regulator of antiviral activity, whose depletion could enhance Sindbis virus replication. Further analyses indicated that depletion of H-Ras results in a robust increase in vesicular stomatitis virus infection and a decrease in Sendai virus (SeV)-induced retinoic acid-inducible gene-I-like receptor (RLR) signaling. Interestingly, however, ectopic expression of wild-type H-Ras results in a biphasic mode of RLR signaling regulation: while low-level expression of H-Ras enhances SeV-induced RLR signaling, high-level expression of H-Ras significantly inhibits this signaling. The inhibitory effects correlate with the activation status of H-Ras. As a result, oncogenic H-Ras, H-RasV12, strongly inhibits SeV-induced IFN-β promoter activity and type I interferon signaling. Conversely, the positive effects exerted by H-Ras on RLR signaling are independent of its signaling activity, as a constitutively inactive form of H-Ras, H-RasN17, also positively regulates RLR signaling. Mechanistically, we demonstrate that depletion of H-Ras reduces the formation of MAVS–TNF receptor-associated factor 3 signaling complexes. These results reveal that the H-Ras protein plays a role in promoting MAVS signalosome assembly in the mitochondria, whereas oncogenic H-Ras exerts a negative effect on type I IFN responses.

## Introduction

Mammalian cells employ multiple pattern-recognition receptors to sense pathogens. Upon pathogen recognition, infected cells rapidly mount innate immune responses, such as the induction of type I interferons (IFN-I) and proinflammatory cytokines and the expression of IFN-stimulated genes (ISGs), to contain and clear pathogens, which also leads to the development of adaptive immune responses (1).

RNA viruses are largely recognized by members of the retinoic acid-inducible gene-I (RIG-I)-like receptor (RLR) family that includes RIG-I, melanoma differentiation-associated gene 5 (MDA5), and laboratory of genetics and physiology 2 (LGP2) (2–6). The RLRs share a DExD/H-box helicase domain and a carboxy-terminal domain (CTD), which are important for ligand RNA binding. RIG-I recognizes viral RNA harboring a 5′-triphosphate moiety, while MDA5 is thought to recognize long double-stranded RNA (7, 8). Both RIG-I and MDA5, but not LGP2, possess two caspase activation and recruitment domains (CARDs) at the N-terminus. Upon binding of viral RNA to the CTD, RIG-I and MDA5 may undergo conformational changes that expose the N-terminal CARDs, which then interact with the CARD of mitochondrial antiviral signaling protein (MAVS/VISA/IPS-1/Cardif) (9–12). MAVS acts as a central platform in the mitochondria. Upon CARD–CARD interaction, MAVS recruits tumor necrosis factor (TNF) receptor-associated factor 3 (TRAF3) and TRAF2/TRAF6. TRAF3 in turn activates downstream TBK1/IKKε kinases and TRAF2/TRAF6 activate TAK1/IKKαβγ kinases, leading to activation of the transcription factors IFN regulatory factor (IRF)3/7 and NF-κB, respectively. IRF3/7 and NF-κB then translocate into the nucleus, where they function cooperatively to induce the production of type I IFNs and proinflammatory cytokines (13, 14).

Ras proteins are small GTP-binding proteins that transduce a wide range of signals to regulate cellular growth, differentiation, and programmed cell death (15). Ras proteins act as molecular switches, cycling between inactive GDP-bound and active GTP-bound conformations (16, 17). Ras–GTP regulates a complex signaling network by binding to distinct classes of effector molecules, such as Raf, phosphatidylinositol 3-kinase, Ral guanine nucleotide-dissociation stimulator, and others (18, 19). Among these signaling pathways, the Raf–MEK–ERK pathway is the best characterized (19). Numerous studies have demonstrated that many cancer cells are susceptible to oncolytic viruses such as vesicular stomatitis virus (VSV) (20, 21), reovirus (22), and Sindbis virus (SBV) (23) due to poor IFN responses in the cancer cells. Activated Ras has been strongly implicated in the impairment of IFN responses in cancer cells (24, 25).

Using a shRNA knockdown strategy to investigate novel molecules involved in IFN responses against SBV infection, we have identified H-Ras, which, however, acted as a positive regulator because depletion of H-Ras by shRNA significantly enhanced SBV replication. We further demonstrated that knockdown of *HRAS* significantly reduced Sendai virus (SeV)-induced IFN-β promoter activity, consistent with antiviral activity assays. Mechanistically, we showed that wild-type H-Ras functioned differently from the oncogenic form of H-Ras, H-RasV12. Our study therefore revealed differential mechanisms exerted by wild-type H-Ras and oncogenic H-Ras in the regulation of IFN responses.

## Materials and Methods

### Plasmid DNAs and shRNAs

The cDNAs coding for human H-Ras and RIG-I was obtained by PCR from HEK293T cells. H-RasV12 and H-RasN17 mutants were then generated using *HRAS* cDNA as a template *via* the PCR site-directed mutagenesis method (26). All wild-type and mutant *HRAS* cDNAs were cloned into the pcDNA3 expression vector. The RIG-I cDNA was cloned downstream of and in-frame with the Flag sequences of the pCMV2-Flag expression vector, whereas pcDNA3-Flag-*MAVS* was provided by Dr. Takashi Fujita (Department of Tumor Cell Biology, Tokyo Metropolitan Institute of Medical Sciences, Japan), pcDNA3.1-Flag-*TBK1* was provided by Dr. John Hiscott (Lady Davis Institute for Medical Research, QC, Canada), and p*IRF3*-5D was provided by Dr. Yi-Ling Lin (Institute of Biomedical Sciences, Academia Sinica, Taiwan). The pIFN-β-Luc (containing the entire IFN-β promoter upstream of firefly luciferase gene) and the pIRF3-Luc (containing the IRF3 responsive element upstream of firefly luciferase gene) reporter plasmids were provided by Dr. Takashi Fujita, and the pNF-κB-Luc reporter plasmid was provided by Dr. Yi-Ling Lin (Institute of Biomedical Sciences, Academia Sinica, Taiwan). The pIFN-stimulated responsive element (ISRE)-Luc plasmid was purchased from Stratagene. Lentiviruses containing control shRNA or shRNAs targeting *HRAS* (#64: CCACCAGTACAGGGAGCAGAT, and #66: GTGTGTGTTTGCCATCAACAA) were purchased from the National RNAi Core Facility, Taiwan. To generate *HRAS* knockdown cells, the cells were infected with shRNA-containing lentiviruses at a multiplicity of infection (MOI) of 5 in the presence of polybrene (8 µg/ml). The infected cells were selected with puromycin (2.5 µg/ml) for 4 days, and the resulting cells were used for subsequent experiments.

### Antibodies

The antibodies used for Western blot include the following: mouse anti-Flag M2 antibody (F3164) and mouse anti-β-actin monoclonal antibody (A5441) from Sigma-Aldrich, rabbit anti-TRAF3 antibody (C-20, H-20) and rabbit anti-IRF3 antibody (sc-9082) from Santa Cruz Biotechnology, rabbit anti-phospho-IRF3 (Ser 386) (ab76493) from Abcam, mouse anti-MAVS (ALX-804-847) from Enzo, and rabbit anti-H-Ras (GTX61164) from Genetex. Antibodies used for immunoprecipitation include the following: mouse anti-Flag M2 antibody (F3164) from Sigma-Aldrich, and rabbit anti-TRAF3 (H-20) from Santa Cruz.

### Viruses, Cell Culture, Transfection, and Luciferase Reporter Assays

Sendai virus and VSV were purchased from ATCC and amplified in the laboratory using embryonated chicken eggs and Vero cells, respectively. HuS-E/2 is an immortalized primary human hepatocyte cell line (27) (kindly provided by Dr. Kunitada Shimotohno, Research Center for Hepatitis and Immunology, National Center for Global Health and Medicine, Chiba, Japan) and was cultivated in Dulbecco’s modified Eagle’s medium (DMEM) supplemented with 20 mM Hepes, 15 μg/ml l-proline, 0.25 µg/ml insulin, 5 × 10^−8^ M dexamethasone, 44 mM NaHCO_3_, 10 mM nicotinamide, 5 ng/ml EGF, 0.1 mM Asc-2P, and 1 µg/ml Plasmocin, whereas HEK293T, HeLa, and Vero cells were cultured in DMEM; all cultures were supplemented with 10% fetal bovine serum (FBS) and penicillin (100 U/ml), streptomycin (0.1 mg/ml), and amphotericin B (0. 25 µg/ml). The cells were cultivated at 37°C with 5% CO_2_.

Plasmid DNAs were transfected into HEK293T cells using the calcium phosphate precipitation method. However, Lipofectamine 2000 (Invitrogen) was used to perform transfections in 96-well plates for reporter assays. To carry out luciferase activity assays from H-Ras knockdown or overexpressed cells, the cells (1 × 10^4^) were transfected in 96-well plates with pIFN-β-Luc, pIRF3-Luc, pNF-κB-Luc, or pISRE-Luc reporter DNA together with the indicated DNAs and the pTK-RL (*Renilla* luciferase) reporter plasmid, which was used as a transfection control. A control vector DNA was added, wherever it was needed, to make the total amount of transfected DNA equivalent in each transfection experiment. Twenty-four hours after transfection, cells were infected with SeV (for transfection with pIFN-β-Luc, pIRF3-Luc, or pNF-κB-Luc) or treated with 1,000 IU/ml IFN-α (for transfection with pISRE-Luc) for 24 h. Cells were assayed using Alamar Blue (AbD Serotec) to measure cell viability, and cell lysates were harvested for luciferase activity using a Dual-Glo Luciferase Assay System (Promega) according to the manufacturer’s instructions. The firefly luciferase activities were first normalized to *Renilla* luciferase activity and to cell viability, and the induction fold was calculated by dividing the normalized Luc values of the SeV-infected (or IFN-α-treated) cells with those of the uninfected (or untreated) cells in each group.

### RT-qPCR

Total cellular RNA was extracted using Trizol reagent, and 1 µg of RNA was reverse transcribed with a random hexamer primer using HiScript I reverse transcriptase (Bionovas Biotechnology Co. Ltd.). One-twentieth of the volume of the cDNA product was then subjected to qPCR with gene specific primers. qPCR was conducted using Fast SYBR Green Master Mix (Thermo Fisher Scientific) according to the manufacturer’s instructions. The induction folds of the investigated genes were calculated as ΔΔCt, by normalizing the values to those of uninfected (or untreated) cells and to glyceraldehyde-3-phosphate dehydrogenase.

### GST–Ras-Binding Domain (RBD) Bead-Based Assays for Activated H-Ras

Activated GTP-bound H-Ras was detected using a Ras activation kit (Abcam, ab128504) according to the manufacturer’s instructions. In brief, cells (6 × 10^5^) transfected with different amounts of the *HRAS-, HRAS*V12-, or *HRAS*N17-expressing plasmid were lysed in 1 ml of ice-cold kit-provided lysis buffer containing protease inhibitors. Fifty microliters of lysate were removed, and 10 µl of 6× Laemmli sample buffer were added; this sample was referred to as the “total Ras load.” The remaining lysates were incubated with 40 µl of GST–RBD fusion protein-linked glutathione Sepharose beads, which had been pre-equilibrated with lysis buffer, at 4°C for 30 min. After centrifugation, the beads were washed once with 1 ml of ice-cold lysis buffer. Fifty microliters of 1× Laemmli sample buffer were added to the samples, which were referred to as “Ras-GTP pulldown” samples. The total Ras load and Ras-GTP pulldown samples were resolved on a 12.5% SDS-PAGE gel. The upper portion of the gel (protein sizes greater than 30 kDa) was stained with Coomassie blue to reveal the amounts of GST–RBD pulled down, whereas the lower part of the gel was processed for Western blot using an anti-H-Ras antibody to detect GTP-bound H-Ras.

### Co-Immunoprecipitation

Cells were lysed on ice for 20 min in lysis buffer containing 1% NP-40, 10 mM Tris–HCl (pH 7.4), 120 mM NaCl, and cocktail protease inhibitor (Roche). The lysates were centrifuged at 13,000 rpm at 4°C for 20 min, and supernatants were recovered for protein quantification with a BCA protein assay kit (Pierce). For immunoprecipitation, 500 µg of lysates were incubated with the indicated antibody at 4°C overnight. Thereafter, 10–20 µl of protein G Sepharose beads (GE Healthcare) were added, and samples were further incubated at 4°C for 2 h. The Sepharose beads were washed twice with ice-cold wash buffer containing 0.2% NP-40, 10 mM Tris–HCl (pH 7.4), and 120 mM NaCl, and heated at 95°C for 5 min in Laemmli sample buffer. After centrifugation, the supernatants were subjected to Western blot analysis.

### VSV Plaque Assay

HEK293T cells (3 × 10^5^) treated with lentiviruses containing control shRNA or shRNAs targeting *HRAS* were infected with VSV at an MOI of 0.001 in serum-free DMEM for 2 h. The medium was then replaced with complete medium containing 10% FBS. Twenty-four hours later, the cell supernatants were harvested, and the VSV titer in the supernatant was determined by plaque assays on Vero cells.

### SBV Replication Assay

An SBV replicon containing a luciferase gene was constructed, and recombinant SBV-Luc virus was produced as previously described (23). HEK293T cells treated with control shRNA or shRNAs targeting *HRAS* were infected with recombinant SBV-Luc virus at an MOI of 30. Forty-eight hours after infection, Luc activity and cell viability were measured using the Bright-Glo Luciferase Assay System (Promega) and an Alamar Blue assay kit, respectively. The luciferase activity was normalized to the Alamar Blue data to reflect SBV-Luc replication activity.

### Statistical Analysis

The data were analyzed by one-way ANOVA followed by Dunnett’s *post hoc* test (Graphpad Prism 5 Software). *** indicates *P* < 0.001, ** indicates *P* < 0.01, and * indicates *P* < 0.05. *P* < 0.05 was regarded as statistically significant.

## Results

### H-Ras Is a Regulator of RNA Virus Replication

We have previously demonstrated that cells exhibiting low susceptibility to infection with SBV, a virus that is extremely sensitive to IFN activity, normally possess intact IFN responses (23). Thus, we rationalized that silencing the expression of molecules that positively regulate IFN responses should render cells more susceptible to SBV infection. By contrast, silencing negative regulators may further inhibit SBV infection in cells. Therefore, we have conducted a high-throughput shRNA screening using a recombinant SBV replicon expressing a luciferase (Luc) gene (SBV-Luc) to screen for novel molecules involved in IFN responses. A mini shRNA library, which targets 123 human oncogenes and tumor suppressor genes, was used for screening in this study. As illustrated in Figure S1A in Supplementary Material, the primary screen identified 57 genes, each of which had at least 2 shRNA clones simultaneously showing the *z*-scores greater than 1 or smaller than −1. All of the shRNAs of these candidate genes were proceeded to the secondary screen using the IFN-β-Luc reporter assay stimulated by N-RIG, the N-terminal 2CARD domain of RIG-I. The secondary screen identified 11 genes, each of which had at least 2 shRNA clones simultaneously exhibiting a twofold or more increase or reduction in the induction fold of IFN-β-Luc activity when compared to the empty vector-transduced cells. Among them, H-Ras was identified (Table S1 in Supplementary Material). Given that the *z*-scores of 7 out of 8 shHRAS clones were positive, albeit some of them were smaller than 1 (Figure S1B in Supplementary Material), we strongly speculated that H-Ras might play a positive role in regulating IFN-I responses. To confirm this thought, we conducted SeV-induced IFN-stimulated responsive element (ISRE)-Luc reporter assay by using the six most effective shHRAS clones based on the *z*-scores. As anticipated, depletion of H-Ras by these shRNA clones significantly reduced the SeV-stimulated ISRE activity (Figure S1C in Supplementary Material). As a result, H-Ras was chosen as a candidate and shHRAS#64 and #66 were used for the following studies.

**Figure 1:**
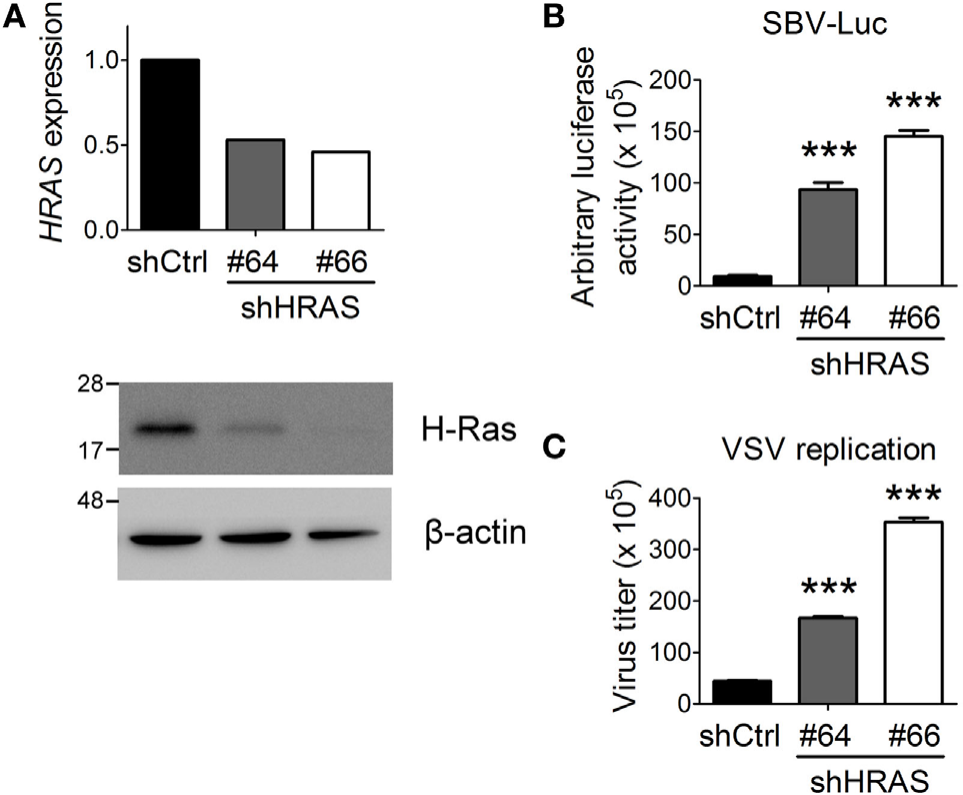
H-Ras depletion enhances the replication of two RNA viruses. **(A)** H-Ras in HEK293T cells was depleted by two independent lentiviral clones, shHRAS#64 and shHRAS#66, which contained shRNAs targeting *HRAS*. RT-qPCR and Western blot were performed to validate the knockdown efficiency of these two shRNAs. **(B)** HEK293T cells were treated with lentiviruses containing control shRNA (shCtrl) or shHRAS#64 or #66 for 4 days. The cells were then infected with a recombinant Sindbis virus containing a luciferase gene (SBV-Luc) at a multiplicity of infection (MOI) of 30. The expression of the luciferase gene was under the control of a subgenomic RNA promoter, which is relied on viral replication. Luciferase activity was measured at 48 h post SBV infection. **(C)** The HEK293T cells depleted of H-Ras by shHRAS#64 or #66 were infected with vesicular stomatitis virus (VSV) at an MOI of 0.001. The medium was harvested at 24 h post-viral infection, and the VSV titer in the supernatant was determined by plaque assays on Vero cells. All data are presented as the mean ± SD of triplicate samples from one representative of at least three independent experiments. ***P < 0.001 between the indicated groups and the control shRNA-treated group (one-way ANOVA followed by Dunnett’s *post hoc* analysis).

First, we confirmed previous screening results by demonstrating that depletion of H-Ras by shRNA #64 or #66 (Figure 1A) led to a robust increase in Luc activity from the SBV replicon (Figure 1B). SBV is a positive-strand RNA virus and belongs to the *Togaviridae* family. To expand these findings, we further examined the effects of H-Ras depletion on infection with VSV, which is a negative-strand RNA virus and is a member of the *Rhabdoviridae* family. Similar to SBV infection, H-Ras depletion increased VSV replication in HEK293T cells as well (Figure 1C). Notably, increased Luc activity from the SBV replicon and increased VSV titers correlated with the decrease in H-Ras protein levels. Therefore, these data demonstrate that H-Ras exerts a positive regulatory role in antiviral activity.

### H-Ras Positively Regulates SeV-Induced RLR Signaling

**Figure 2:**
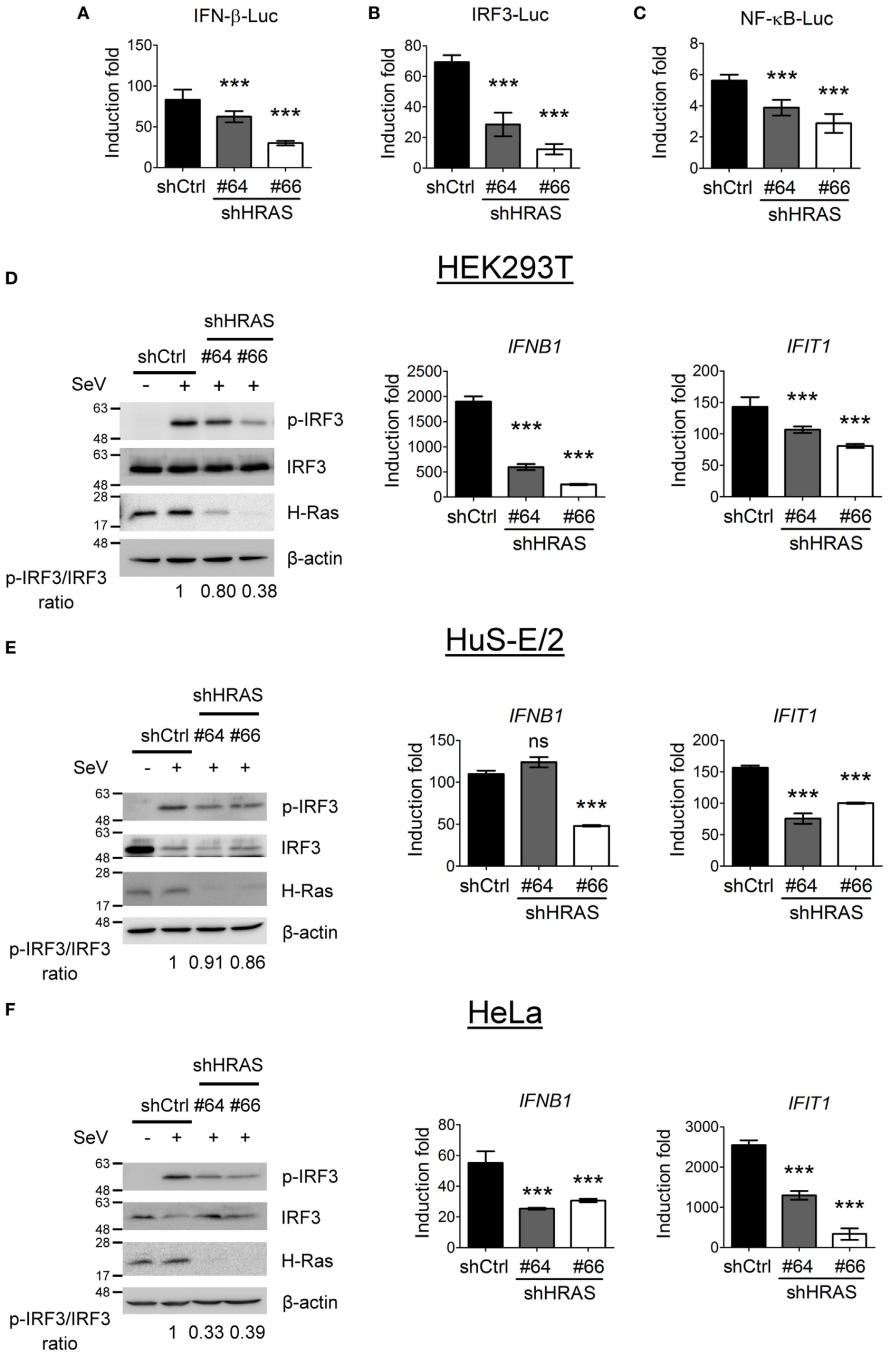
H-Ras positively regulates Sendai virus (SeV)-induced RLR signaling. **(A–C)** The HEK293T cells treated with control shRNA (shCtrl) or depleted of H-Ras by shHRAS#64 or #66 were transfected with the pIFN-β-Luc reporter plasmid **(A)**, the pIRF-3-Luc reporter plasmid **(B)**, or the pNF-κB-Luc reporter plasmid **(C)** together with a TK-*Renilla* luciferase reporter plasmid (used as a transfection control). The cells were mock infected or infected with 20 HAU/ml SeV at 24 h post-transfection, and luciferase activities were measured at 24 h post-infection. Firefly luciferase activities were first normalized to *Renilla* luciferase activity, and the induction folds were calculated by further normalizing the Luc activity of the infected cells to those of the uninfected cells in each knockdown group. **(D)** The HEK293T cells, **(E)** HuS-E/2, or **(F)** HeLa cells treated with control shRNA or depleted of H-Ras by shHRAS#64 or #66 were mock infected or infected with SeV (20 HAU/ml). The levels of IRF3 phosphorylation were analyzed at 24 h post-infection by Western blot using a rabbit anti-phospho-IRF3 (Ser386) antibody, whereas the gene transcription levels of *IFNB1* and *IFIT1* were examined by RT-qPCR. The ratios of phospho-IRF3 to total IRF3 are shown at the bottom of each Western blot and the induction folds of RNA transcripts were calculated as ΔΔCt, by normalizing the values of infected cells to those of uninfected cells and to glyceraldehyde-3-phosphate dehydrogenase. All data are presented as the mean ± SD from three independent experiments. ***P < 0.001 between the indicated groups and the control shRNA-treated group (one-way ANOVA followed by Dunnett’s *post hoc* analysis). ns, not significant.

To investigate the mechanism underlying the antiviral activity of H-Ras, we used these two shRNAs to further conduct reporter assays in HEK293T cells to understand whether H-Ras regulated the RLR signaling induced by SeV infection. The results showed that shRNA-mediated silencing of H-Ras significantly decreased the SeV-induced activities of IFN-β-Luc (Figure 2A), IRF-3-Luc (Figure 2B), and NF-κB-Luc (Figure 2C), suggesting that H-Ras indeed positively regulated RLR signaling. Consistently, H-Ras silencing reduced the SeV-induced IRF3 phosphorylation, leading to reduced RNA expression of downstream genes such as *IFNB1* and *IFIT1* (Figure 2D). To understand whether this phenomenon is specific to HEK293T cell only, we also examined the effect of *HRAS* knockdown on RLR signaling in two other cell lines. Similarly, in HuS-E/2 cells, an immortalized primary human hepatocyte cell line (Figure 2E) or in HeLa cells (Figure 2F), *HRAS* knockdown also led to reduced IRF3 phosphorylation albeit a little less in HuS-E/2 cells and reduced *IFNB1* and *IFIT1* gene expression upon SeV infection. Collectively, these data confirm a positive regulatory role of H-Ras in IFN-I responses and demonstrate that the stimulatory effect is not limited to a particular cell type.

### Wild-type H-Ras Regulates RLR Signaling in a Biphasic Mode Depending on H-Ras Activation Levels

**Figure 3:**
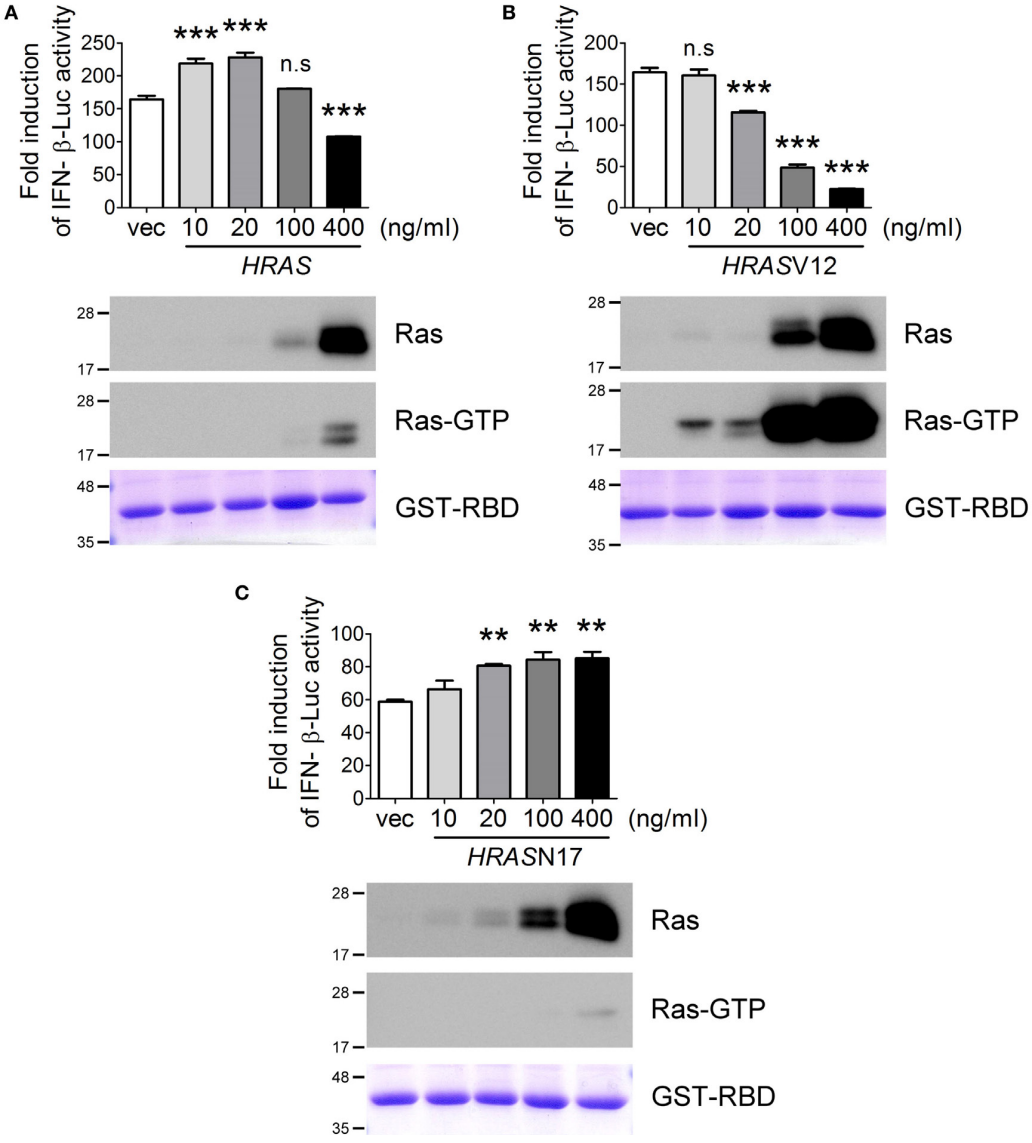
The H-Ras protein enhances RLR signaling, whereas H-Ras oncogenic activity inhibits RLR signaling. HEK293T cells were transfected with the wild-type *HRAS* DNA **(A)**, the *HRAS*V12 DNA **(B)**, or the *HRAS*N17 DNA **(C)** at the indicated transfection doses together with the pIFN-β-Luc reporter plasmid and the pTK-RL reporter plasmid. In all groups, the total amounts of transfected DNA were adjusted to the same using the control vector DNA. Twenty-four hours post-transfection, cells were infected with Sendai virus (SeV) (20 HAU/ml). Luciferase activities were measured at 24 h post-infection. The results of induction fold were obtained from three independent experiments and presented as the mean ± SD. **P < 0.01 and ***P < 0.001 between the indicated groups and the control vector-transfected group (one-way ANOVA followed by Dunnett’s *post hoc* analysis). ns, not significant. The expression levels of transfected *HRAS* were detected by Western blot, shown below the bar chart. To analyze the activation status of the ectopically expressed H-Ras, we conducted another experimental set and cell lysates were harvested 24 h post SeV infection. Ras activation status was examined using GST–Ras-binding domain (RBD) Sepharose beads according to the manufacturer’s instructions. The proteins bound on the beads were separated on a 12.5% SDS-PAGE gel. The upper part of the gel (MW > 30 kDa) was stained with Coomassie blue to reveal the amount of GST–RBD precipitated (the bottommost panels), whereas the lower part of the gel (MW < 30 kDa) was processed for Western blot using a rabbit anti-H-Ras antibody to show the GTP-bound H-Ras, Ras-GTP.

Next, we examined the effects of ectopic expression of *HRAS* on SeV-induced RLR signaling using the IFN-β-Luc reporter assay. Surprisingly, we found that H-Ras protein influenced SeV-induced IFN-β promoter activity in a biphasic manner rather than strictly enhancing promoter activity as we originally anticipated (Figure 3A). The ectopically expressed H-Ras enhanced IFN-β promoter activity in a dose-dependent manner at low transfection doses, e.g., 10 and 20 ng/ml. However, IFN-β promoter activity was gradually inhibited by increasing the transfection dose to 100 or 400 ng/ml. Using the GST-fused RBD of Raf as an activation-specific probe for the detection of Ras-GTP (28) to reveal the activation status of ectopically expressed H-Ras in the cells, we found that H-Ras was activated to a certain extent when it was expressed to an extreme high level (Figure 3A).

As reported previously, oncogenic Ras exerts negative effects on IFN-I responses (24, 25). Thus, we speculated that the inhibition of SeV-induced IFN-β promoter activity at high levels of wild-type H-Ras might be caused by the negative effects of H-Ras activation on IFN responses. To confirm this, we examined SeV-stimulated IFN-β-Luc activity in the presence of ectopically expressed H-RasV12, which is the constitutively GTP-bound form of Ras, or H-RasN17, which is the constitutively GDP-bound form. The results demonstrated that, indeed, H-RasV12 inhibited SeV-induced IFN-β-Luc activity even at a low transfection dose such as 20 ng/ml, and that the inhibition was strongly correlated with the levels of activated Ras (Figure 3B). Conversely, H-RasN17 increased SeV-induced IFN-β-Luc activity in a dose-dependent manner (Figure 3C). Consistent with its constitutive negative activity, the activation-specific probe revealed minimal levels of GTP-bound Ras even when *HRAS*N17 DNA was transfected at a high dose, e.g., 400 ng/ml. These results thus exclude the possibility that the inhibitory effects of wild-type H-Ras transfected at 400 ng/ml dose was due to a competitive expression between the transfected reporter DNA and *HRAS* DNA, but support for the argument that the H-Ras protein itself rather than its activity exerts a positive role, whereas activated H-Ras exerts a negative role in regulating RLR signaling. As a result, wild-type H-Ras, when expressed at low levels, functions in a manner similar to H-RasN17, which enhances RLR signaling. However, H-Ras becomes partially activated when artificially expressed to high levels, which resembles H-RasV12 and inhibits RLR signaling.

### The H-Ras Protein Acts on Signaling Molecules Upstream of IRF3

**Figure 4:**
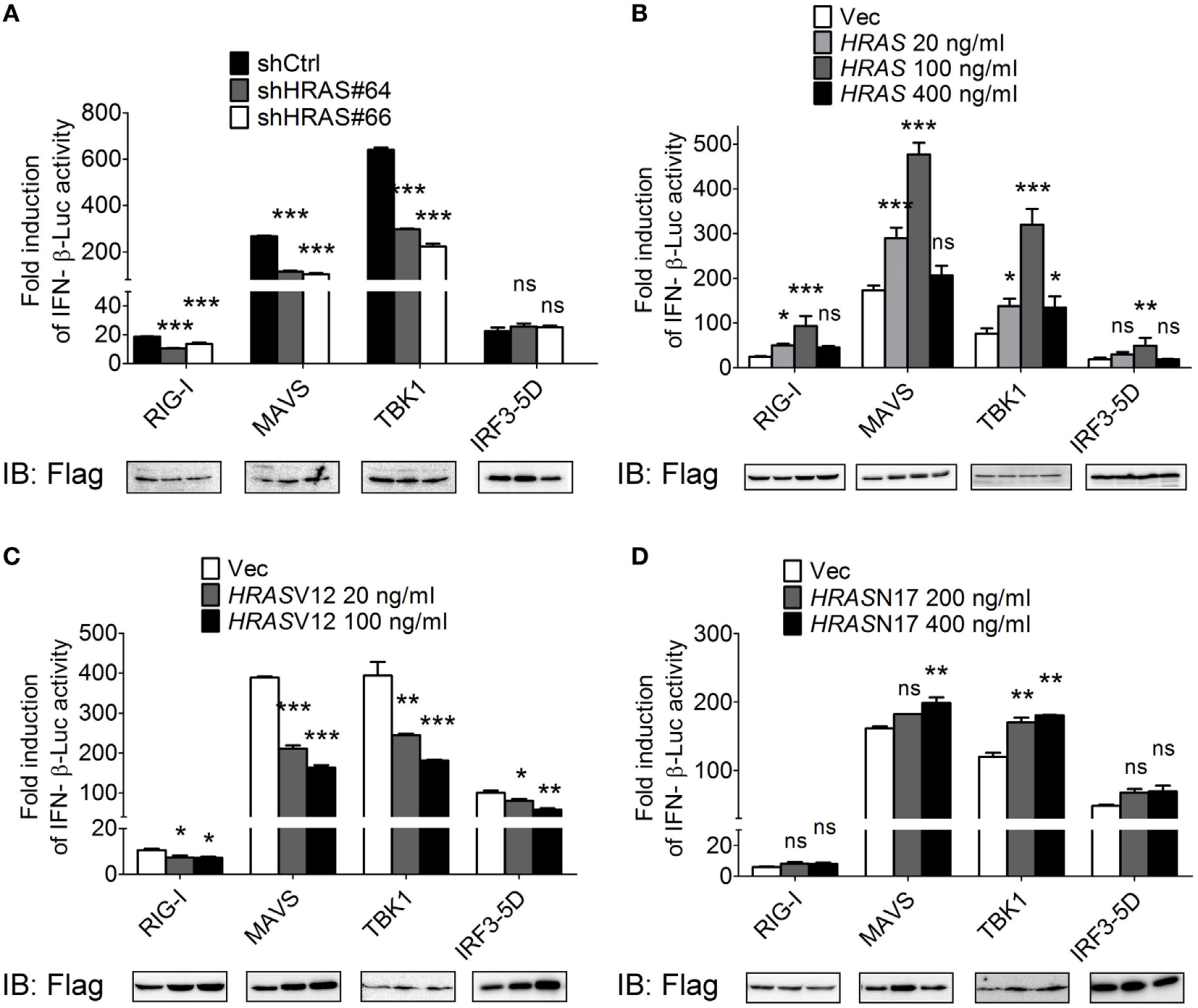
H-Ras protein acts on the RLR signaling pathway upstream of IRF3. HEK293T cells were depleted of H-Ras by shHRAS#64 or #66 **(A)**, or transfected with wild-type *HRAS* **(B)**, *HRAS*V12 **(C)**, or *HRAS*N17 **(D)** at the indicated doses. The cells were also transfected with the pIFN-β-Luc reporter plasmid and the pTK-RL reporter plasmid together with the Flag-tagged retinoic acid-inducible gene-I (RIG-I)-, MAVS-, TBK1-, or IRF3-5D-expressing plasmids to induce the RLR signaling. In all groups, the total amounts of transfected DNA were adjusted to the same using the control vector DNA. Luciferase activities were measured at 24 h post-transfection. The expression levels of each transfected inducer molecule were revealed by Western blot shown below the bar chart. The results of induction fold were obtained from three independent experiments and presented as the mean ± SD. *P < 0.05, **P < 0.01, and ***P < 0.001 between the indicated groups and the shCtrl-treated or the vector-transfected group (one-way ANOVA followed by Dunnett’s *post hoc* analysis). ns, not significant.

To dissect the step at which H-Ras acts to regulate the RLR signaling pathway, we expressed the Flag-tagged individual molecules in the signaling pathway, such as RIG-I, MAVS, TBK1, or IRF3-5D (a constitutively active form of IRF3), to induce the signaling and IFN-β-Luc activity under *HRAS* knockdown condition or in the presence of ectopically expressed wild-type H-Ras, H-RasV12, or H-RasN17 was determined. The constitutively active form of IRF3 was used because IRF3 *per se* would not be activated in the absence of upstream activated TBK1. The reporter assay data showed that H-Ras depletion significantly reduced IFN-β promoter activity induced by all inducers except IRF3-5D (Figure 4A). Ectopically expressed wild-type H-Ras exhibited a similar biphasic mode of regulation on the IFN-β-Luc activity mediated by all inducers (Figure 4B), whereas ectopically expressed H-RasV12 significantly inhibited IFN-β-Luc activity mediated by all inducers (Figure 4C). H-RasN17 demonstrated a lower effect and only upregulated MAVS- and TBK1-induced IFN-β-Luc activity (Figure 4D). Under all of the induction conditions, the expression levels of each inducer were more or less comparable within each experimental set, arguing against the possibility that the stimulatory or inhibitory effects were due to differential expression levels of the inducers. These data suggest that the H-Ras protein may function at a step upstream of IRF3, likely involving the mitochondria-associated platform. Conversely, the oncogenic H-Ras may affect not only the RLR pathway but also the signaling downstream of the RLR pathway, e.g., IFN signaling as previously reported (24, 25, 29).

### *HRAS* Knockdown Reduces the Formation of the MAVS–TRAF3 Signaling Complex

**Figure 5:**
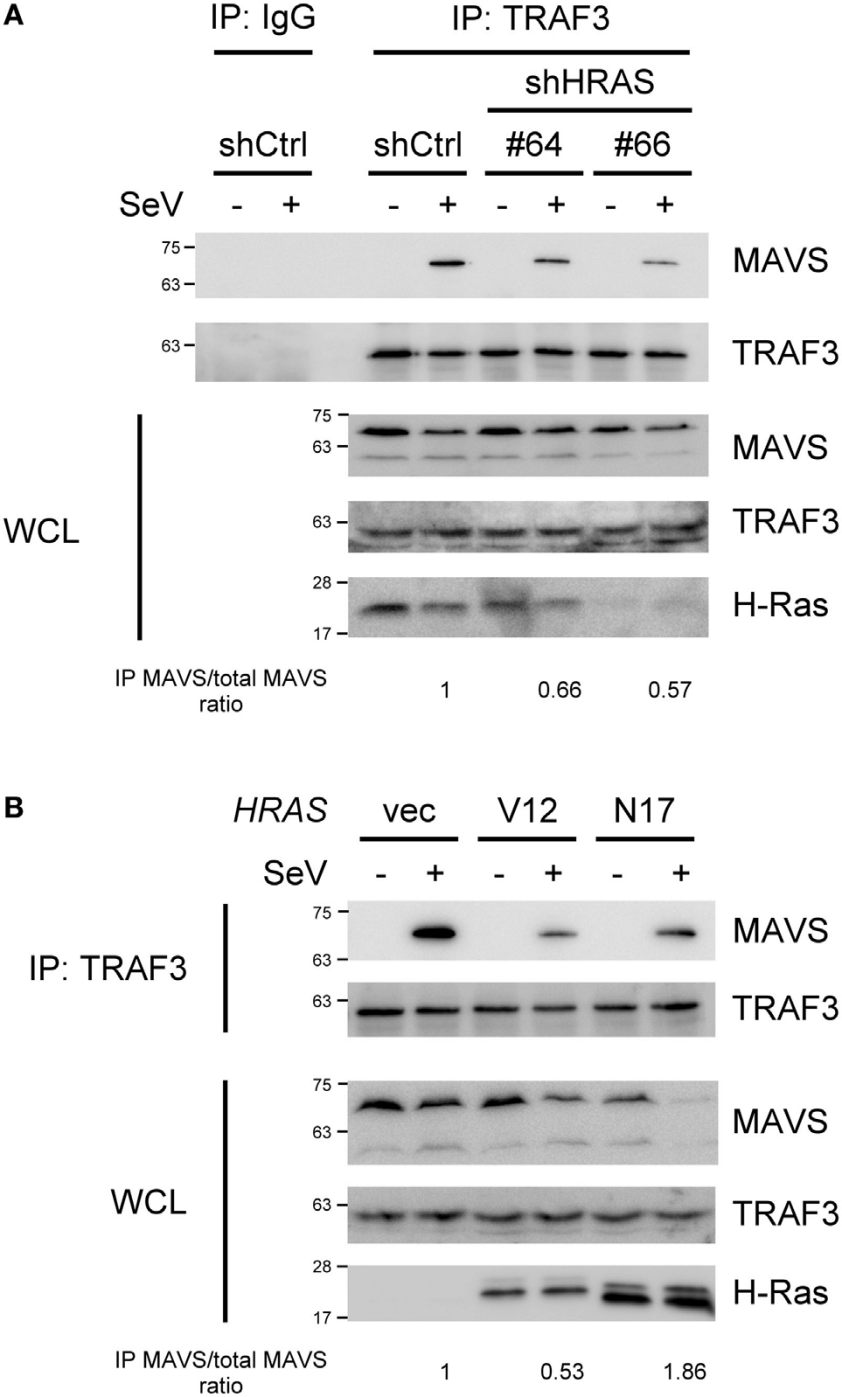
H-Ras and H-RasN17 enhances the formation of the MAVS–TNF receptor-associated factor 3 (TRAF3) signaling complex, whereas H-RasV12 reduces it. **(A)** HEK293T cells were depleted of H-Ras by shHRAS#64 or #66, or **(B)** cells were transfected with vector, the *HRAS*V12 DNA (15 ng/ml), or the *HRAS*N17 DNA (60 ng/ml) at the indicated doses shown in the parenthesis. In all groups, the total amounts of transfected DNA were adjusted to the same using the control vector DNA. After knockdown or DNA transfection, the cells were then mock infected or infected with Sendai virus (SeV) (20 HAU/ml) for 24 h. Cell lysates were immunoprecipitated with an anti-TRAF3 antibody or control IgG antibody, followed by Western blot analysis using anti-MAVS and anti-TRAF3 antibodies. WCL, whole cell lysate. The ratios of immunoprecipitated MAVS relative to total MAVS are shown at the bottom.

Since H-Ras functions at a step upstream of IRF3, we hypothesized that the H-Ras protein might participate in the MAVS-associated platform, as a subpopulation of H-Ras proteins localize to the membranes of the ER, Golgi, endosome, and mitochondria (30–32). To understand whether H-Ras could possibly associate with any components within the MAVS-associated signalosome, we expressed H-Ras together with Flag-tagged RIG-I, MAVS, TBK1, or TRAF3 in HEK293T cells, and co-immunoprecipitation experiments were performed. However, none of these proteins were found to interact with H-Ras (data not shown). We further examined whether H-Ras played a role in the formation of the MAVS-associated signalosome. The formation of MAVS–TRAF3 complex stimulated by SeV infection was detected by co-immunoprecipitation experiments under H-Ras depletion conditions. As shown in Figure 5A, we were able to detect significant amounts of MAVS interacting with TRAF3 in the cells treated with control shRNA, upon SeV infection but not in the absence of viral infection. However, MAVS–TRAF3 interaction was reduced with the depletion of H-Ras by shHRAS#64 or #66. It was noteworthy that the total levels of MAVS proteins in the whole cell lysate were all discernibly reduced upon SeV infection, a phenomenon that has also been reported previously by Castanier et al. (33). In their study, they have found that the activation of RLR signaling induces the formation of MAVS-associated signalosome, which in turn leads to a proteasomal degradation of MAVS induced by TRIM25-mediated ubiquitination, thus freeing TBK1 to phosphorylate IRF3 (33). Hence, we preferred to use the ratios of immunoprecipitated MAVS/total MAVS, shown at the bottom of each knockdown group, to more accurately reflect the efficiency of the MAVS–TRAF3 complex formation. The results indicated that the depletion of H-Ras indeed reduced the formation of the MAVS–TRAF3 complex.

We also investigated the formation of the MAVS–TRAF3 complex under ectopic expression of H-RasV12 or H-RasN17. However, since the doses of RasV12 and RasN17 to exhibit their maximal effects on RLR signaling were different, the DNA amounts used for transfection of each molecule were titrated individually and different amounts of DNA were transfected here. As shown in Figure 5B, at the optimal effective dose, H-RasV12 was found to significantly suppress the MAVS–TRAF3 complex formation. H-RasN17 also seemed to reduce the apparent levels of MAVS–TRAF3 complex when compared to the control vector, but the total MAVS levels in the whole cell lysates were also significantly reduced, indicating an active transduction of the signal. Thus, the ratio of immunoprecipitated MAVS/total MAVS was actually increased, suggesting an increased efficiency of the MAVS–TRAF3 complex formation, by ectopic expression of H-RasN17. The results were consistent with those of reporter assays shown in Figures 3 and 4.

### Oncogenic H-Ras Negatively Regulates IFN-I Signaling

**Figure 6:**
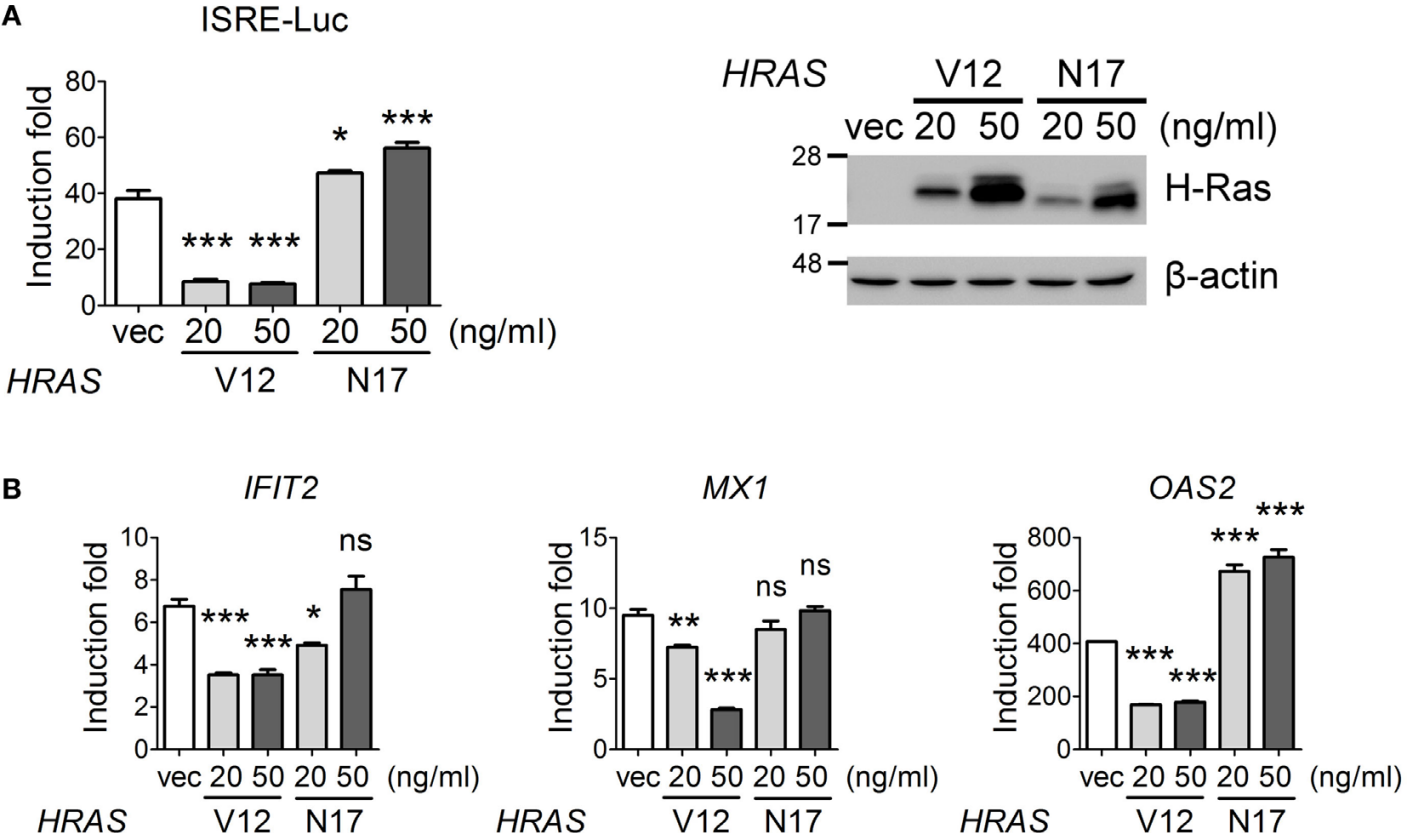
Oncogenic H-Ras inhibits type I interferon signaling. **(A)** HEK293T cells were transfected with vector, *HRAS*V12, or *HRAS*N17 DNA at the indicated doses together with the pISRE-Luc reporter plasmid and the pTK-RL reporter plasmid. In all groups, the total amounts of transfected DNA were adjusted to the same using the control vector DNA. Cells were treated with or without IFN-α (1,000 U) at 24 h post-DNA transfection, and luciferase activities were measured at 24 h post IFN-α treatment. The expression levels of transfected H-Ras are shown on the right. **(B)** The RNA transcription levels of *IFIT2, MX1*, and *OAS2* were determined by RT-qPCR and the induction folds were calculated as ΔΔCt. All the results of induction fold were obtained from three independent experiments and presented as the mean ± SD. *P < 0.05, **P < 0.01, and ***P < 0.001 between the indicated groups and the vector-transfected group (one-way ANOVA followed by Dunnett’s *post hoc* analysis).

On the other hand, we would also like to confirm that oncogenic H-Ras downregulated IFN signaling pathway. We first carried out an IFN-stimulated responsive element (ISRE)-Luc reporter assay under IFN-α treatment conditions in the presence of H-RasV12 or H-RasN17 (used as a control). The results showed that H-RasV12 significantly inhibited, whereas H-RasN17 slightly upregulated, the IFN-α-induced ISRE promoter activity (Figure 6A). RT-qPCR analysis also indicated that H-RasV12 inhibited the expression of several ISGs downstream of IFN signaling, such as *IFIT2, MX1*, and *OAS2*, whereas H-RasN17 had minimal effects on or even enhanced the expression of these ISGs (e.g., *OAS2*) (Figure 6B).

We tried to investigate the mechanisms underlying the inhibitory activity of H-RasV12 on IFN-I signaling by examining whether H-RasV12 affected the activation levels of STAT1 and STAT2. However, no inhibition of IFN-α-stimulated STAT1 or STAT2 phosphorylation by H-RasV12 or by H-RasN17 was observed (Figure S2A in Supplementary Material). The total amounts of STAT1 or STAT2 proteins were also not reduced, either at 30 min or at 24 h after IFN-α treatment (Figure S2A in Supplementary Material). Thus, our data did not support the mechanism reported previously, in which H-RasV12 downregulated IFN signaling by reducing the total amounts of STAT2 (25). We further examined whether H-RasV12 influenced the translocation of the activated STAT1/STAT2 complex into the nucleus. The cytosolic and nuclear fractions of *HRAS*V12- or *HRAS*N17-transfected cells were separated at 1 h post IFN-α treatment, and phospho-STAT1 levels were determined by Western blot. However, we still did not observe significant differences in the translocation of phospho-STAT1 between control cells and cells expressing H-RasV12 or H-RasN17 (Figure S2B in Supplementary Material). Thus, although our data demonstrate that oncogenic H-Ras indeed impairs IFN-I signaling, the mechanism still remains elusive.

### H-Ras Also Stimulates IFN-I Responses Mediated by MDA5 or STING Signaling, but Does Not Influence TNF-α, IL-6, or IFN-γ Signaling

**Figure 7:**
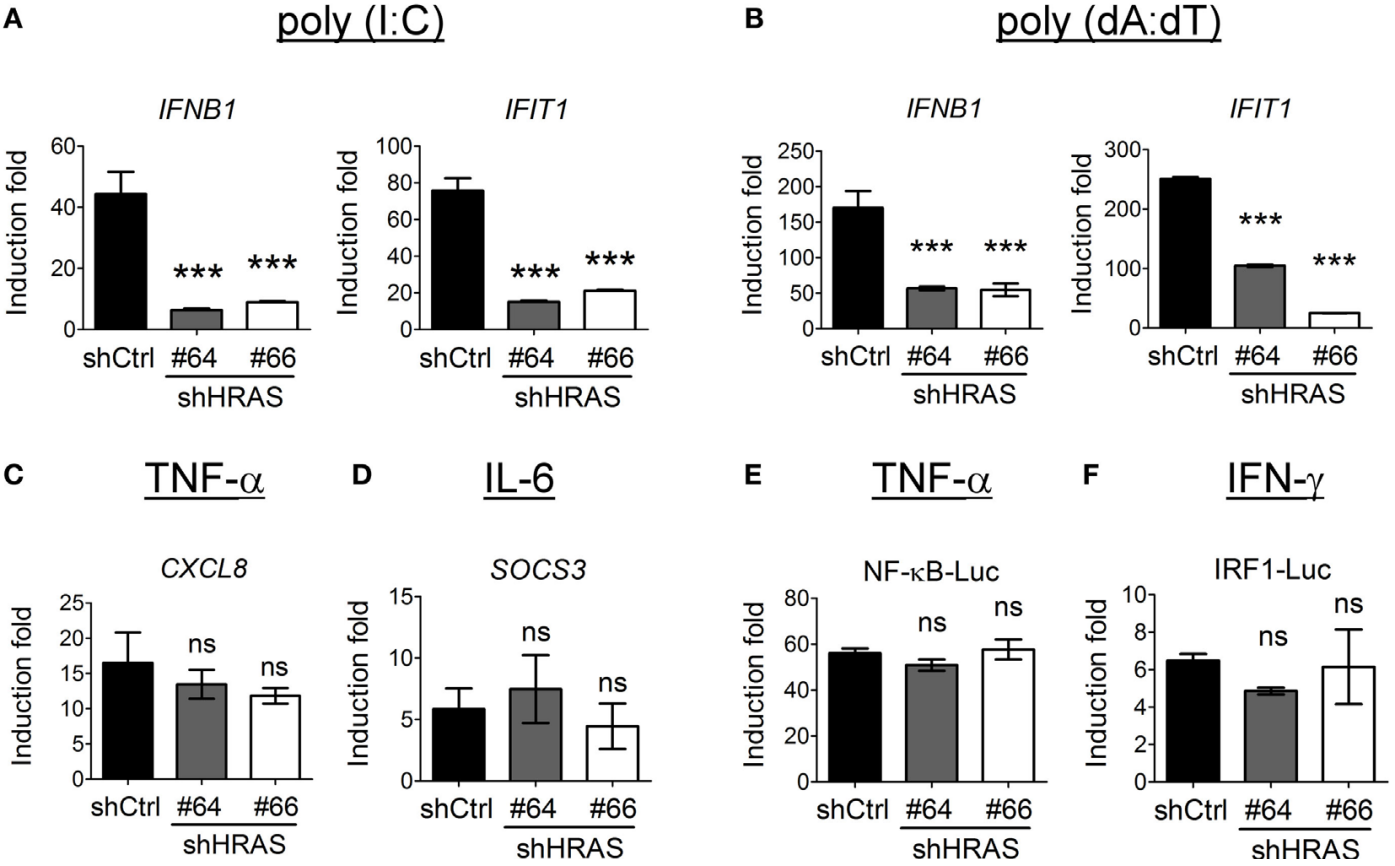
H-Ras regulates melanoma differentiation-associated gene 5 (MDA5)- and STING-mediated type I interferon responses but does not influence tumor necrosis factor (TNF)-α, IL-6, or IFN-γ signaling. HEK293T cells were treated with control shRNA (shCtrl) or shRNAs targeting *HRAS* (#64 or #66). **(A)** The cells were first transfected with the MDA5-expressing plasmid for 24 h, then transfected with poly(I:C) (1 µg/ml) for 8 h to induce MDA5 signaling, or **(B)** they were transfected with poly(dA:dT) (20 µg/ml) for 8 h to induce STING signaling. Gene transcription of *IFNB* and *IFIT1* was analyzed by RT-qPCR. **(C,D)** The shRNA-treated HEK293T cells were treated with TNF-α (40 ng/ml) **(C)**, or with IL-6 (40 ng/ml) **(D)**, for 18 h. The downstream gene transcription of *CXCL8* and *SOCS3*, respectively was analyzed by RT-qPCR. **(E,F)** The shRNA-treated HEK293T cells were transfected with pNF-κB-Luc reporter plasmid **(E)** or pIRF1-Luc reporter plasmid **(F)**, together with the pTK-RL plasmid. In all groups, the total amounts of transfected DNA were adjusted to the same using the control vector DNA. Cells were treated with TNF-α (40 ng/ml) **(E)**, or with IFN-γ (500 ng/ml) at 24 h post-DNA transfection, and luciferase activity was measured at 18 h post cytokine treatment. The results of induction fold were obtained from three independent experiments and presented as the mean ± SD. ***P < 0.001 between the indicated groups and the control shRNA-treated group (one-way ANOVA followed by Dunnett’s *post hoc* analysis). ns, not significant.

In addition to the enhancement of RIG-I-mediated signaling by H-Ras, we sought to verify whether H-Ras also enhanced IFN responses induced by MDA5- or STING (stimulator of interferon genes)-mediated signaling. For studying MDA5 signaling, we first transfected the HEK293T cells that were treated with control shRNA or shRNAs targeting *HRAS* with MDA5 cDNA since HEK293T cells express extremely low levels of MDA5 (23), and subsequently transfected them with long-chain poly(I:C) to induce MDA5 signaling. To induce STING signaling, the shRNA-treated HEK293T cells were transfected with poly(dA:dT). Expression of *IFNB1* and *IFIT1* RNA in response to ligand stimulation was analyzed by RT-qPCR. The results showed that depletion of H-Ras significantly downregulated the transcription of these two genes induced by poly(I:C) (Figure 7A) or by poly(dA:dT) (Figure 7B), indicating that H-Ras also positively regulates the IFN-I responses induced by MDA5 or STING signaling. On the other hand, depletion of H-Ras did not affect the TNF-α-induced *CXCL8* expression (Figure 7C), or the IL-6-induced *SOCS3* expression (Figure 7D), indicating that *HRAS* knockdown did not influence TNF-α or IL-6 signaling. Alternatively, luciferase reporter assays also demonstrated that depletion of H-Ras had no effects on the TNF-α-induced NF-κB-Luc activity (Figure 7E) or the IFN-γ-induced IRF1-Luc activity (Figure 7F), further indicating that H-Ras also did not influence IFN-γ signaling. Taken together, these results strongly suggest that H-Ras exhibits specific effects on IFN-I responses, and ruled out the possibility that the observed suppressive effects on IFN-I responses by H-Ras knockdown was due to a reduced cell proliferation effect.

## Discussion

In this study, we report that H-Ras can modulate IFN-I antiviral immunity in two different ways, depending on the activation status of the H-Ras protein. First, gene disruption strategies revealed that H-Ras exerted a positive regulatory role in antiviral activity, evidenced by biochemical analyses showing that RIG-I-, MDA5-, and STING-mediated IFN-I responses were attenuated when H-Ras was depleted. Mechanistically, we demonstrated that H-Ras enhances SeV-induced RLR signaling by promoting MAVS–TRAF3 signaling complex formation. Notably, the positive regulatory role of H-Ras is independent of its signaling activity. Second, we found that oncogenic H-Ras or overexpressed wild-type H-Ras significantly attenuated IFN-I signaling and SeV-stimulated IFN-β promoter activity. However, the underlying mechanisms of this attenuation remain elusive. Collectively, these data reveal the unexpected roles of H-Ras in the regulation of IFN-I antiviral immune responses.

The Ras proteins undergo a series of post-translational modifications, such as farnesylation, carboxymethylation, and palmitoylation, at their C-terminal hypervariable regions after protein synthesis (30–32), which results in the addition of membrane trafficking motifs and confers membrane-binding affinity on the proteins. Therefore, Ras protein can access the membranes of many different cellular organelles, such as the plasma membrane, ER, Golgi, endosome, and mitochondria. van Zuylen et al. recently reported that the Golgi apparatus can disorganize into fragmented compartments that contain TRAF3 upon viral infection. These membrane-bound vesicles then contact the mitochondrial membranes to form the mitochondria-associated membranes (MAMs), facilitating the proper positioning and interaction of TRAF3 with MAVS and leading to the activation of TBK1 and IRF3 (34). Given that a subpopulation of Ras proteins localize to the membranes of the ER/Golgi or mitochondria, it is tempting to speculate that Ras proteins may facilitate MAM formation by acting as interorganellar bridges, where the H-Ras protein on the ER/Golgi interacts with yet-to-be identified proteins on the mitochondria, or *vice versa*, leading to the enhancement of MAM formation and MAVS signalosome assembly. The importance of H-Ras in MAVS signalosome formation was evidenced by our observation that the interaction between TRAF3 and MAVS was significantly reduced when H-Ras was depleted (Figure 5A). The scenario may also apply to poly(dA:dT)-stimulated STING signaling pathway as STING is an ER-localized transmembrane protein and plays an essential role in DNA sensing signaling pathway, leading to IFN-I production (35). Upon activation, STING relocalizes to perinuclear puncta where it interacts with TBK1, leading to phosphorylation of IRF3 (36). Therefore, we hypothesize that the juxtaposition of these small membrane-bound vesicles may also be facilitated by the endomembranous Ras proteins.

Our results showed that H-Ras knockdown significantly downregulated IFN-I responses, including the RIG-I-, MDA5-, and STING-mediated signaling (Figures 7A,B), but did not affect TNF-α, IL-6, or IFN-γ signaling (Figures 7C–F). We reason that H-Ras may function on the mitochondria- or the ER-associated membranes to facilitate signalosome assembly, but has no effect on cytokine receptors that localize on cell surface. It is well documented that the plasma membrane-associated Ras proteins are highly involved in a wide range of signals to regulate cell growth, differentiation, and programmed cell death (15), which all require the GTPase activity of Ras proteins. Thus, the plasma membrane-associated Ras may function differently from the endomembranous Ras in regulating different signaling.

Previous studies have demonstrated that oncogenic Ras can prevent IFN-I responses, rendering many Ras-transformed cancer cells susceptible to oncolytic virus replication (20–23). The data reveal that activation of the signaling pathway downstream of Ras, specifically the Raf–MEK–ERK pathway, severely compromises IFN-β production (37) and type I IFN signaling (24, 25, 38). In mouse NIH3T3 cells and L929 cells stably expressing constitutively activated Ras (RasV12), the levels of phospho-STAT1, phospho-STAT2, and total amounts of STAT2 were all reduced (25). However, this phenomenon was not observed in our *HRAS*V12-transfected HEK293T cells, despite the fact that the expression of downstream IFN signaling ISGs was significantly inhibited (Figure 6). We reasoned that this could be attributable to the different cell lines used in different experiments and that other mechanisms may also underlie the impairment of IFN responses by oncogenic Ras. Ahn et al. recently reported that Ras transformation results in the cleavage of the reticulon protein Nogo-B, which can suppress IFN responses. However, this mechanism is distinct from the activated MEK/ERK pathway (29). Therefore, it is likely that in addition to Raf–MEK–ERK signaling, different signaling pathways or cellular factors can be modulated by activated Ras signaling and are involved in the impairment of IFN responses. Further studies must delineate the mechanisms underlying the downregulation of IFN responses in *HRAS*V12-transfected HEK293T cells.

Taken together, our study provides evidence showing that the H-Ras protein can enhance the assembly of the MAVS-associated signalosome upon viral infection in normal cells, thus exerting a positive role in antiviral activity. This event is independent of H-Ras signaling activity. However, the activated Ras suppresses IFN-I responses through diverse mechanisms, which can render the Ras-transformed cancer cells susceptible to virus replication. It has been demonstrated that the bridges linking the ER and the mitochondria are composed of high-molecular weight protein complexes (39), and that upon virus infection, MAVS aggregates to form a prion-like structure that is required for the recruitment of downstream TRAF molecules (40, 41). Thus, defining the components within these huge MAVS signalosomes specifically at MAM sites will provide new insights to our understanding of antiviral signaling.

## Author Contributions

G-AC conducted most of the experiments, analyzed the results, and wrote the basic manuscript. Y-RL conducted the experiments of poly(I:C) and poly(dA:dT) transfection analysis. H-TC firstly identified the positive regulatory role of H-Ras in RLR signaling and conducted antiviral activity assays. L-HH conceived the idea of the project, support G-AC, Y-RL, and H-TC in all aspects of the experiments, and finish the final version of the manuscript.

## Conflict of Interest Statement

The authors declare that the research was conducted in the absence of any commercial or financial relationships that could be construed as a potential conflict of interest.
