# Supplementary material for: H-Ras Exerts Opposing Effects on Type I Interferon Responses Depending on Its Activation Status
Source: Front Immunol. 2017 Aug 11;8:972. doi: 10.3389/fimmu.2017.00972 (PMC5554495; doi:10.3389/fimmu.2017.00972)
Supplement: Supplementary file 1 [file data_sheet_1.pdf]

Supplementary materials

Methods

shRNA screen

A mini lentiviral library, which was comprised of 838 shRNA clones targeting 123 human oncogenes and tumor suppressor genes, was used for the screen. These VSV-G pseudotyped lentiviruses, containing several shRNAs targeting different sequences of the same RNA transcript, were from the RNAi Consortium (TRC) and the mini library was prepared by the RNAi Core, Taiwan. Hep3B cells seeded in 384-well plates (1,000 cells/well) were infected with the shRNA-containing lentiviruses in triplicates with one shRNA clone per well. The transduced cells were selected with puromycin (2.5 μg/ml) for four days until the mock-infected or uninfected cells were completely dead. The survived cells were subsequently infected with SBV-Luc virus at an MOI of 30. Two days after Sindbis virus infection, cells were harvested for Alamar Blue assay to determine the cell viability and cell lysates were used for luciferase activity assay. The luciferase activities were first normalized to the cell viability, and SBV replication folds were calculated by dividing the normalized Luc values of the shRNA-transduced cells with those of the empty vector-transduced cells. The log values of the replication folds were converted to z-scores using the following formula: $z = (x - \mu)/\sigma$, where x represents the log fold, μ represents the mean, and σ represents the standard deviation, of the 838 clones.

To reduce the numbers of shRNA acting through an off-target effect, we selected the candidate genes which had at least two shRNA hits simultaneously showing the z-scores greater than 1 or smaller than -1. After the first round of screening, all the shRNAs of the candidate genes were performed the secondary screening using the IFN-β-Luc reporter assay stimulated by N-RIG (the N-terminal 2CARD domain of RIG-I). HEK293T cells (1×10^4 cells) seeded in 96-well plates were infected with the shRNA-containing lentiviruses and then selected with puromycin for four days. The survived cells were then transfected with pIFN-β-Luc and pTK-RL, together with or without the N-RIG-expressing plasmid. Twenty-four hours after DNA transfection, cells were harvested for Alamar Blue assay and cell lysates were used for luciferase activity assay. The firefly luciferase activity was normalized to *Renilla* luciferase activity and to cell viability; the induction folds were calculated by dividing the normalized Luc values of the N-RIG-transfected cells with those of the untransfected cells.

Fractionation of cytosolic and nuclear proteins

Cytosolic and nuclear proteins were fractionated following a previously described protocol (1). In brief, cells (6×10^5) were washed once with ice-cold PBS and scrapped into micro-centrifuge tube. After a pop-spin on a benchtop centrifuge for 10 s, supernatants were removed and cell pellets were resuspended in 300 μl of 0.1% NP40 in PBS with 5 times pipetting. One hundred microliters of lysate were removed, and 33 μl of 4x Laemmli buffer was added; this sample was referred to as the "whole cell lysate". The remaining lysates were pop-spin again for 10 s, 100 μl of supernatants were removed, and 33 μl of 4x Laemmli buffer was added; this sample was referred to as the "cytosolic fraction". After removing all remaining supernatants, the pellets were washed once with ice-cold 0.1% NP40 in PBS and then resuspended

in 60 µl of 1x Laemmli buffer; this fraction was referred to as the “nuclear fraction”. The whole cell lysate and the nuclear fraction were further sonicated with microprobe to disrupt DNA contents. Finally, all the samples in Laemmli buffer were boiled for 5 min and then resolved on a 12.5% SDS-PAGE gel, followed by western blot analysis.

1. Suzuki, K., Bose, P., Leong-Quong, R.Y., Fujita, D.J., and Riabowol, K. (2010). REAP: A two minute cell fractionation method. *BMC Res Notes* 3**,** 294. doi: 10.1186/1756-0500-3-294.

Supplementary figures

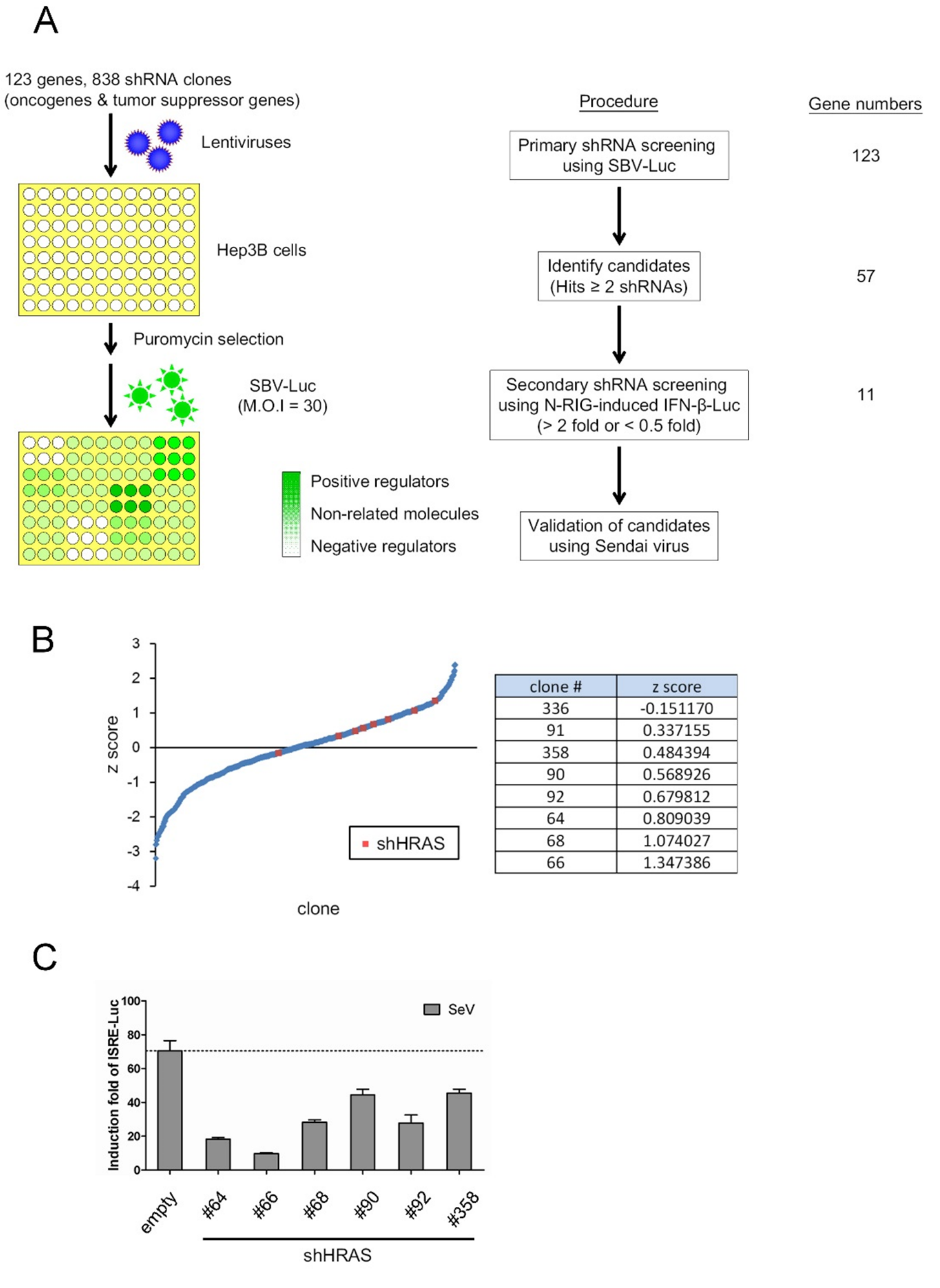

clone #	z score
336	-0.151170
91	0.337155
358	0.484394
90	0.568926
92	0.679812
64	0.809039
68	1.074027
66	1.347386

Figure S1. shRNA screen identifies candidate oncogenes or tumor suppressor genes which may regulate IFN-I responses.
(A) Schematic representation of shRNA screen and outline of the screen procedure. The numbers of genes associated with each screen process are shown on the right side. **(B)** The z-score curve for all of the shRNAs is illustrated. The red dots and the numbers in the table indicate the z-scores for the eight shHRAS clones. **(C)** HEK293T cells depleted of H-Ras by shHRAS #64, #66, #68, #90, #92, or #358 were transfected with pISRE-Luc and pTK-RL reporter plasmids. In all groups the total amounts of

transfected DNA were adjusted to the same using the control vector DNA. Cells were mock infected or infected with 20 HAU/ml SeV at 24 h post DNA transfection. Twenty-four hours after SeV infection, cells were harvested for Alamar Blue assay and cell lysates were used for luciferase activity assay. The firefly luciferase activities were first normalized to *Renilla* luciferase activity and to cell viability, and the induction folds were calculated by further dividing the normalized Luc activity of the infected cells with that of the uninfected cells in each knockdown group.

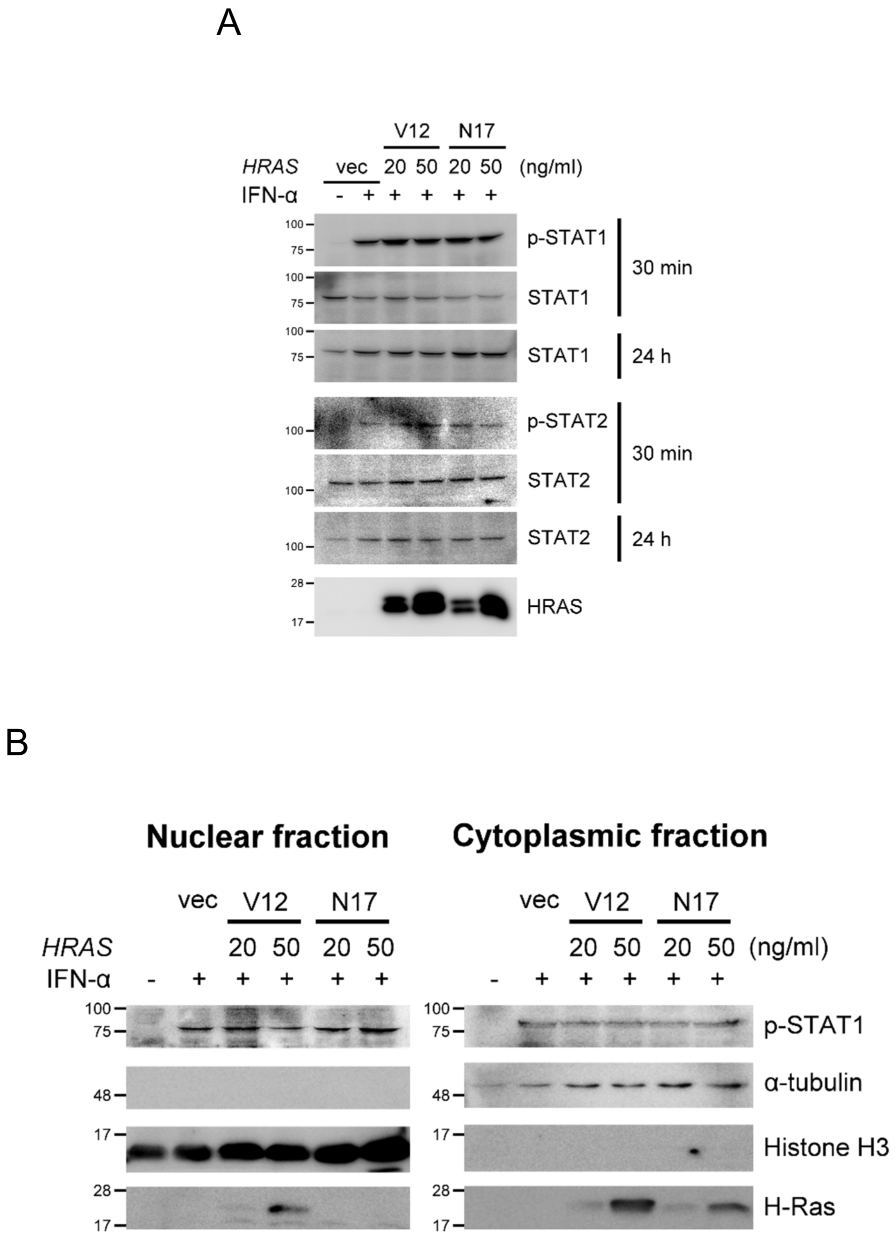

Figure S2. H-RasV12 does not inhibit STAT1/STAT2 phosphorylation or nuclear translocation upon IFN-α treatment.

(A) HEK293T cells were transfected with vector, *HRAS*V12, or *HRAS*N17 DNA at the indicated doses. Cells were treated with IFN-α (1,000 U) at 24 h post-transfection, and the phosphorylation levels of STAT1 and STAT2 were examined at 30 min post IFN-α treatment using rabbit anti-phospho-STAT1 (Y701) and rabbit anti-phospho-STAT2 (Y690) antibodies, respectively. Total STAT1 and STAT2 protein levels were also examined by western blot at 30 min and 24 h post IFN-α treatment. **(B)** HEK293T cells were transfected with vector, *HRAS*V12, or *HRAS*N17 DNA at the indicated doses. Proteins from the nuclear fraction or the cytoplasmic fraction were separated. The levels of phospho-STAT1 in each fraction were detected by anti-phospho-STAT1 antibody. The presence of tubulin or histone H3 was used to indicate the cytoplasmic and the nuclear fractions, respectively, and also to indicate the purity of each fractionation.

Supplementary table

Table S1. Hit lists of genes identified in the screens

Gene	Primary screening ≥ 2 hits	Secondary screening > 2 fold or < 0.5 fold
ABL1	✓	✓ (-) [1]
AKT2		
APC		
BCL2	✓	
BCL3	✓	
BCR		
BRCA1	✓	
BRCA2		
CBL		
CCND1		
CD82		
CDH1		
CDK2AP2		
CDK4	✓	✓ (-)
CDKN1A	✓	
CDKN1C	✓	
CDKN2A		
CDKN2B		
CRK		
CSF1R		
DCC		
E2F1	✓	
EGFR	✓	
ELK1		
ELK3		
EPHA1		
EPHA3		
ERBB2	✓	✓ (-)
ERBB3		
ERBB4	✓	
ERG		

Gene	Primary screening ≥ 2 hits	Secondary screening > 2 fold or < 0.5 fold
ETS1	✓	
ETS2	✓	
FAT		
FAT2	✓	
FAT3		
FER	✓	
FES		
FGF3		
FGF4		
FGF6	✓	
FGR		
FLI1	✓	
FOS		
FOSL1	✓	
FOSL2	✓	✓ (+)
FYN		
GLTSCR1	✓	
GLTSCR2		
HCK	✓	✓ (+)
HRAS	✓	✓ (+)
JUN	✓	
JUNB	✓	
JUND		
KIT		
KRAS	✓	
LATS1	✓	
LATS2		
LCK	✓	
LOC339951		
LOC401525		
LYN		
LZTS1		
LZTS2		
MAS1		

Gene	Primary screening ≥ 2 hits	Secondary screening > 2 fold or < 0.5 fold
MAX	✓	
MCC		
MCF2		
MET	✓	✓ (-)
MLH1	✓	
MOS		
MSH2	✓	
MTUS1	✓	
MYB		
MYBL1	✓	
MYBL2		
MYC	✓	
MYCL1		
MYCN		
NBL1	✓	✓ (+)
NF1	✓	
NF2		
NRAS		
NTRK1	✓	✓ (-)
OVCA2	✓	
PDGFB	✓	
PIM1		
PTCH	✓	
RAF1	✓	
RB1	✓	
REL		
RET	✓	
ROS1		
SKI		
SMAD4	✓	
SRC		
ST13	✓	
ST14		
ST18		

Gene	Primary screening ≥ 2 hits	Secondary screening > 2 fold or < 0.5 fold
ST5		
ST7		
ST7L		
TAL1	✓	
TGFBR2	✓	
THRA	✓	
THRB		
TIAM1		
TP53		
TSSC1	✓	
TSSC4	✓	
TUSC1		
TUSC2		
TUSC3		
TUSC4		
TUSC5	✓	
UNK	✓	
VAV1		
VHL	✓	✓ (+)
VHLL	✓	
WNT1	✓	
WNT2		
WT1	✓	
YES1	✓	✓ (-)
**Total (123)**	**57**	**11**

[1] (+) and (-) indicate that the proteins displayed positive or negative effects, respectively on the N-RIG-induced IFN-β-Luc activity.
